# A New Disease

**Published:** 1891-08

**Authors:** 


### A New Disease.

Two English physicians—Dr. Hale White and Mr. Golding-Bird—have recently described an affection to which they give the name "Idioglossia." It Appears that the patients hear well, and express themselves in articulture sounds, but such sounds are unlike those of any known language. The patients really have a language entirely of their own, in which there does not seem to be any confusion, *i. e.*, the sounds given forth have an intellgent application, and the same sound always has the same meaning. The discussion before the Royal Medical and Chirurgical Society was varied, some of the members contending that the so-called language of those affected was but a modification of the English tongue, and was to be accounted for by a lack of development in that particular direction.—*Exchange.*

